# Korean red ginseng extract prevents bone loss in an oral model of glucocorticoid induced osteoporosis in mice

**DOI:** 10.3389/fphar.2024.1268134

**Published:** 2024-03-12

**Authors:** Nicholas J. Chargo, Ho Jun Kang, Subhashari Das, Yining Jin, Cheryl Rockwell, Jae Youl Cho, Laura R. McCabe, Narayanan Parameswaran

**Affiliations:** ^1^ Department of Physiology, Michigan State University, East Lansing, MI, United States; ^2^ College of Osteopathic Medicine, Michigan State University, East Lansing, MI, United States; ^3^ Department of Pharmacology and Toxicology, Michigan State University, East Lansing, MI, United States; ^4^ Department of Integrative Biotechnology, Sungkyunkwan University, Suwon, Republic of Korea; ^5^ College of Human Medicine, Michigan State University, East Lansing, MI, United States

**Keywords:** Korean red ginseng extract, glucocorticoid, bone, microbiota, gut-bone axis, gut barrier, immune populations

## Abstract

The gut microbiota and barrier function play important roles in bone health. We previously demonstrated that chronic glucocorticoid (GC)-induced bone loss in mice is associated with significant shifts in gut microbiota composition and impaired gut barrier function. Korean Red Ginseng (KRG, *Panax Ginseng* Meyer, Araliaceae) extract has been shown to prevent glucocorticoid-induced osteoporosis (GIO) in a subcutaneous pellet model in mice, but its effect on gut microbiota and barrier function in this context is not known. The overall goal of this study was to test the effect of KRG extract in a clinically relevant, oral model of GIO and further investigate its role in modulating the gut-bone axis. Growing male mice (CD-1, 8 weeks) were treated with 75 μg/mL corticosterone (∼9 mg/kg/day) or 0.4% ethanol vehicle in the drinking water for 4 weeks. During this 4-week period, mice were treated daily with 500 mg/kg/day KRG extract dissolved in sterile water or an equal amount of sterile water via oral gastric gavage. After 4 weeks of treatment, we assessed bone volume, microbiota composition, gut barrier integrity, and immune cells in the bone marrow (BM) and mesenteric lymph nodes (MLNs). 4 weeks of oral GC treatment caused significant distal femur trabecular bone loss, and this was associated with changes in gut microbiota composition, impaired gut barrier function and altered immune cell composition. Importantly, KRG extract prevented distal femur trabecular bone loss and caused significant alterations in gut microbiota composition but had only modest effects on gut barrier function and immune cell populations. Taken together, these results demonstrate that KRG extract significantly modulates the gut microbiota-bone axis and prevents glucocorticoid-induced bone loss in mice.

## Introduction

Glucocorticoids (GC) are potent anti-inflammatories used to treat or manage inflammation and inflammatory diseases such as rheumatoid arthritis, systemic lupus erythematosus, and inflammatory bowel disease (Vandewalle et al., 2018; Hardy et al., 2020). It is estimated that more than 3 million people in the United States are currently undergoing chronic GC therapy (Overman et al., 2013), and the number has likely increased with the COVID-19 pandemic (Goel et al., 2021). Although GCs have excellent clinical efficacy and provide patients with symptomatic relief, there are several unwanted side effects associated with their use over time. These side effects include increased body weight, elevated plasma glucose, muscle wasting, and of particular interest to our study, bone loss (Yasir et al., 2023; Gensler, 2012; Gado et al., 2022). Bone loss from chronic glucocorticoid use (or glucocorticoid-induced osteoporosis, GIO) is the leading cause of secondary osteoporosis (i.e., bone loss caused by another disease or treatment of another disease) (Briot and Roux, 2015). Clinically, GIO leads to a significant increase in fracture risk and raises the Fracture Risk Assessment (FRAX) score (Kanis et al., 2011; Amiche et al., 2016; 2018; Lee et al., 2020).

Although there are some effective treatment options for GIO, adherence is often poor due to unwanted side effects, inconvenience of daily medications/injections, and the cost of treatment (Buckley et al., 2017; Adami and Saag, 2019b; 2019a; Lane, 2019; Iacopo et al., 2020; Lim and Yeap, 2022). As such, there is a need for novel therapies that confer little to no side effects, are convenient for patients to use consistently, and are cost effective. Recent studies from our lab and others have shown that gut microbiota plays a critical role in regulating bone health in several etiologies of bone loss (Sjögren et al., 2012; McCabe et al., 2015; Weaver, 2015; Hernandez, 2017; Quach and Britton, 2017; Wallace et al., 2017; Ohlsson and Sjögren, 2018; Pacifici, 2018; Cooney et al., 2020; Jeyaraman et al., 2023). We demonstrated that the microbiota composition is altered and linked with bone loss in several mouse models including GIO, post-antibiotic dysbiosis, type-1 diabetes, estrogen deficiency, and obesity (Britton et al., 2014; McCabe et al., 2019; Schepper et al., 2019; 2020; Rios-Arce et al., 2020; Kang et al., 2023). Importantly, in these mouse models of bone loss, treatment with probiotics, particularly *Lactobacillus reuteri* 6475 significantly prevented bone loss. Additionally, our lab and others have shown that gut barrier dysfunction is linked to bone loss. Treatment with probiotics not only modulates the gut microbiota, but also prevents gut barrier dysfunction and therefore, prevents bone loss (Li et al., 2016; Schepper et al., 2019; 2020; Behera et al., 2021). These findings intricately link the composition of the gut microbiota and gut barrier function to bone health in several etiologies of osteoporosis.

Recently we showed that Korean Red Ginseng (KRG, *Panax Ginseng* Meyer, Araliaceae) extract was effective at preventing bone loss in a post-antibiotic dysbiosis model in mice (Kang et al., 2023). We also demonstrated that KRG extract treatment altered gut microbiota composition as well as improved gut barrier function in the dysbiosis model. Based on these studies we wanted to test if KRG extract treatment can prevent bone loss in a clinically relevant oral GC-induced bone loss model in mice and whether the gut-bone axis was important for these effects. Although previous studies have shown a protective role for KRG extract in preventing GIO in a subcutaneous GC pellet model (Kim et al., 2015), the role of gut microbiota and barrier function were not assessed. In this study, we show that KRG extract prevents trabecular bone loss and significantly modulates the composition of the gut microbiota at the species level in GIO. Its effect on gut permeability, however, was modest. Our studies underscore the usefulness of KRG extract in modulating gut microbiota and preventing bone loss that occurs with chronic GC treatment.

## Materials and methods

### Korean Red Ginseng

**FIGURE 1:**
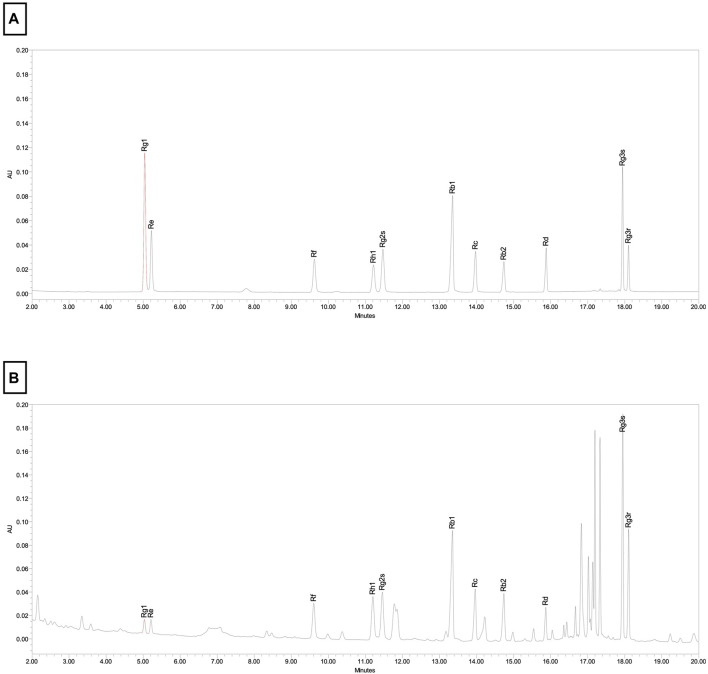
Chromotographic fingerprint of KRG extract. **(A)** HPLC results from ginsenoside standards present in KRG extract. **(B)** HPLC results from KRG extract.

Korean red ginseng (KRG, *Panax Ginseng* Meyer, Araliaceae) extract was provided by Korea Ginseng Corporation (Daejeon, Korea) and its major metabolites as previously reported (Kim et al., 2021) are shown in Table 1. Briefly, KRG extract was prepared in a 75:25 ratio of main roots to lateral or fine roots via a hot water extraction and concentration process as described previously (Lee et al., 2015; So et al., 2018). A compositional fingerprint of the KRG extract ginsenosides was completed by HPLC. Briefly, HPLC analysis was carried out on Waters HPLC system and a UV detector (Waters 2487, Milford, MA, United States of America), as previously reported (Kang et al., 2009; Kim et al., 2013). Separations were performed on a Sun fire C18 column (3.5 μm, 4.6 × 150 mm, Waters). The elution conditions were as follows: solvent A, water; solvent B, acetonitrile; gradient, 0–22 min (18% B), 22–32 min (18%–30% B), 32–60 min (30%–50% B). The flow rate was 1 mL/min and detected at the UV wavelength of 203 nm. 20 μL of KRG extract were injected and each peak was identified by comparing their retention times with that of each reference ginsenoside. Chromatogram of ginsenoside standards is shown in Figure 1A and a representative ginsenoside fingerprint of the extract used in this study is shown in Figure 1B.

**TABLE 1:** Korean Red Ginseng extract major metabolites.

Metabolite	Amount/g
Ginsenoside-Rg1	5.5 mg
Ginsenoside-Rg2	5.5 mg
Ginsenoside-Rg3	5.5 mg
Carbohydrates	0.33 g

### Animals and experimental design

All animal procedures were approved by the Michigan State University (MSU) Institutional Animal Care and Use Committee and conformed to NIH guidelines. Seven-week-old male CD-1 mice were purchased from Charles River Laboratories (Wilmington, MA, United States) and allowed to acclimate to the MSU vivarium for 1 week prior to beginning experiments. Animals were housed at four to five mice per cage, on a 12:12 h light-dark cycle, and had *ad libitum* access to sterilized standard chow (Teklad 2019; Teklad, Madison, WI, United States) and water. At 8 weeks of age, mice were randomly assigned to a treatment group and treated with the glucocorticoid (GC) Corticosterone (CS) (Sigma 27840) at 75 μg/mL (∼9 mg/kg/day) or 0.4% ethanol (EtOH) vehicle in the drinking water. KRG extract was dissolved in sterile water and was administered daily at 500 mg/kg/day in 100 μL via oral-gastric gavage for 4 weeks. Controls were gavaged daily with an equal volume of water. Groups for the 4-week experiment were as follows: 1. Vehicle + Vehicle (0.4% EtOH + water), 2. Vehicle + KRG extract (0.4% EtOH +500 mg/kg/day KRG extract), 3. GC + Vehicle (75 μg/mL CS + water), 4. GC + KRG extract (75 μg/mL CS + 500 mg/kg/day KRG extract). Cages were changed, body weights recorded, and food and water intake were measured weekly. Mice were fasted 4 h prior to euthanasia so plasma glucose could be recorded with a Metene blood glucose monitoring system (TD-4116, Metene Ltd., Walnut, CA, United States). Mice were euthanized via inhaled isoflurane overdose followed by cervical dislocation, an American Veterinary Medical Association approved euthanasia method. One mouse in the GC + KRG extract group died during the experiment due to unknown causes, however no other mice experienced any health issues. Experiments were done in two different cohorts of mice for a total of n = 17–18 mice/group. For some assays (where indicated in the figure legends) only one cohort was utilized for analysis as indicated, due to sample limitations.

### Microcomputed tomography (μCT) bone analysis

Femurs and vertebrae were collected during harvest, fixed in 10% formalin, and scanned in a GE Explore Locus μCT (GE Healthcare, Piscataway, NJ, United States) at a resolution of 20 μm obtained from 720 views and were analyzed as previously described (Rios-Arce et al., 2020; Schepper et al., 2020). A fixed threshold of 600 was used for all analyses. Trabecular bone was analyzed in a region of the distal femur defined as 10% of the total bone length proximal to the distal growth plate and excluded cortical bone. Cortical bone was analyzed in a 2 × 2 × 2 mm box at the midshaft of the femur. We also analyzed trabecular bone within the L4 vertebral body. Trabecular bone parameters including volume, thickness, spacing, and number and cortical bone parameters including thickness, area, periosteal perimeter, endocortical perimeter, and moment of inertia were obtained using GE Healthcare Microview software version 2.2.

### *In vivo* whole intestinal permeability

Whole intestinal permeability was assessed using 4kD fluorescein isothiocyanate dextran (FITC-dextran) as previously described (Schepper et al., 2019; Kang et al., 2023). Briefly, mice were gavaged with 300 mg/kg FITC dissolved in sterile PBS at least 4 h prior to time of death. Sterile blood was collected via direct cardiac puncture immediately after death and serum was collected after blood clotted at room temperature for 10 min and was then chilled on ice. Serum fluorescence was measured using a Tecan Infinite M 1000 fluorescent plate reader (Tecan, Mannedorf, Switzerland) at a wavelength of 485/530 nm. The rate of FITC transfer to the serum was then calculated against a standard curve and normalized to the average of the vehicle group.

### Microbiota analyses

Colonic fecal samples from the first cohort of animals (n = 10/group) were processed and analyzed with the ZymoBIOMICS^®^ Targeted Sequencing Service (Zymo Research, Irvine, CA). The following description was written and provided by ZymoBIOMICS^®^.

**DNA Extraction:** One of three different DNA extraction kits was used depending on the sample type and sample volume. In most cases, the ZymoBIOMICS®-96 MagBead DNA Kit (Zymo Research, Irvine, CA) was used to extract DNA using an automated platform. In some cases, ZymoBIOMICS^®^ DNA Miniprep Kit (Zymo Research, Irvine, CA) was used. For low biomass samples, the ZymoBIOMICS^®^ DNA Microprep Kit (Zymo Research, Irvine, CA) was used as it permits for a lower elution volume, resulting in more concentrated DNA samples.

**Targeted Library Preparation:** Bacterial 16S ribosomal RNA gene targeted sequencing was performed using the Quick-16S™ NGS Library Prep Kit (Zymo Research, Irvine, CA). In most cases, the bacterial 16S primers amplified the V3-V4 region of the 16S rRNA gene. These primers have been custom-designed by Zymo Research to provide the best coverage of the 16S gene while maintaining high sensitivity. The sequencing library was prepared using an innovative library preparation process in which PCR reactions were performed in real-time PCR machines to control cycles and therefore limit PCR chimera formation. The final PCR products were quantified with qPCR fluorescence readings and pooled together based on equal molarity. The final pooled library was cleaned with the Select-a-Size DNA Clean & Concentrator™ (Zymo Research, Irvine, CA), then quantified with TapeStation^®^(Agilent Technologies, Santa Clara, CA) and Qubit^®^ (Thermo Fisher Scientific, Waltham, WA).

**Control Samples:** The ZymoBIOMICS^®^ Microbial Community Standard (Zymo Research, Irvine, CA) was used as a positive control for each DNA extraction. The ZymoBIOMICS^®^ Microbial Community DNA Standard (Zymo Research, Irvine, CA) was used as a positive control for each targeted library preparation. Negative controls (i.e., blank extraction control, blank library preparation control) were included to assess the level of bioburden carried by the wet-lab process.

**Sequencing:** The final library was sequenced on Illumina^®^ MiSeq™ with a v3 reagent kit (600 cycles). The sequencing was performed with 10% PhiX spike-in.

**Bioinformatics Analysis:** Unique amplicon sequences variants were inferred from raw reads using the DADA2 pipeline (Callahan et al., 2016). Potential sequencing errors and chimeric sequences were also removed with the Dada2 pipeline. Chimeric sequences were also removed with the DADA2 pipeline. Taxonomy assignment was performed using Uclust from Qiime v.1.9.1 with the Zymo Research Database, a 16S database that is internally designed and curated, as reference. Composition visualization, alpha-diversity, and beta-diversity analyses were performed with Qiime v.1.9.1 (Caporaso et al., 2010). If applicable, taxonomy that have significant abundance among different groups were identified by linear discriminant analysis effect size (LEfSe) (Segata et al., 2011) using default settings. Other analyses such as heatmaps, Taxa2ASV Deomposer, and PCoA plots were performed with internal scripts.

**Absolute Abundance Quantification:** A quantitative real-time PCR was set up with a standard curve. The standard curve was made with plasmid DNA containing one copy of the 16S gene and one copy of the fungal ITS2 region prepared in 10-fold serial dilutions. The primers used were the same as those used in Targeted Library Preparation. The equation generated by the plasmid DNA standard curve was used to calculate the number of gene copies in the reaction for each sample. The PCR input volume was used to calculate the number of gene copies per microliter in each DNA sample. The number of genome copies per microliter DNA sample (genome_copies) was calculated by dividing the gene copy number by an assumed number of gene copies per genome. The value used for 16S copies per genome is 4. The amount of DNA per microliter DNA sample (DNA_ng) was calculated using an assumed genome size of 4.64 × 106 bp, the genome size of *Escherichia coli*, for 16S samples. This calculation is shown below:

Calculated Total DNA = Calculated Total Genome Copies × Assumed Genome Size (4.64 × 10^6^ bp) × Average Molecular Weight of a DNA bp (660 g/mole/bp) ÷ Avogadros Number (6.022 × 10^23^/mole)

For further analyses, raw reads were imported into Qiita (Gonzalez et al., 2018), a GUI interface for QIIME2, were demultiplexed, trimmed to 250bp, deblurred to generate and identify amplified sequence variants, and then assigned taxonomy by the Silva database. Further analysis included generation of principal coordinate analysis plots from distance matrices generated from weighted unifrac distances and PERMANOVA testing for between group differences.

### Immune cell population analyses

Following euthanasia, bone marrow and mesenteric lymph nodes were collected and processed as described before (Steury et al., 2018). Briefly, mesenteric lymph nodes (MLNs) were homogenized, treated with red blood cell lysis buffer, filtered through 40 μm nylon mesh, and counted for flow cytometry analysis. For bone marrow cells, after isolation of total bone marrow cells (both nucleated and non-nucleated cells) from mouse femur, cells were processed and resuspended in media for further flow cytometry analysis as previously described (Schepper et al., 2020).

**Flow cytometry:** Flow cytometry analysis was done as described previously (Lee et al., 2013). Briefly, processed cells were incubated with fc-gamma-R blocking antibody (anti-mouse CD16/32) to block non-specific binding and were then surface-stained with antibody cocktail and washed with staining buffer (phosphate-buffered saline with sodium azide and bovine calf serum). The antibodies used against cell surface markers were CD3 AF488, CD4 PE-Cy7, CD8 Percp-cy5.5, CD19 PE and Nk1.1 BV421. All antibodies were obtained from Biolegend and used as per the manufacturer’s instructions. All cells were run on Attune NxT and the data were analyzed with FlowJo software.

### Statistical analyses

Statistical analyses were performed using GraphPad Prism software version 9 (GraphPad, San Diego, CA, United States). Group differences for body parameters, bone data, gut permeability, and immune cell populations were analyzed via One-Way ANOVA with Tukey’s posttest. Group differences in specific microbiota was analyzed using Kruskall-Wallis One-Way ANOVA with Dunn’s multiple comparison posttest. Range of data are shown as violin plots with lines at the median and quartiles unless otherwise specified. All analyses were completed blinded to treatment group.

## Results

### General body parameters

Eight-week-old male CD-1 mice received glucocorticoid (GC) corticosterone (75 μg/mL) or 0.4% ethanol vehicle in sterile drinking water for 4 weeks. There was no difference in water intake between vehicle and GC treated groups at any time point (data not shown). All mice were gavaged daily with 500 mg/kg Korean Red Ginseng (KRG) extract dissolved in 100 μL sterile water or an equal volume of sterile water (vehicle). As shown in Table 2, at the end of the experiment, body weight, liver and total kidney weights did not differ between groups. Spleen weight was significantly reduced with GC treatment (GC + Vehicle) and KRG extract treatment (GC + KRG extract) did not affect this decrease (*p* < 0.0001, compared to vehicle group). KRG extract alone did not affect spleen weight. Visceral fat was significantly increased with GC treatment (GC + Vehicle group) (*p* = 0.0044, compared to vehicle group) and KRG extract treatment (GC + KRG extract group) modestly decreased GC’s effect. Fasting blood glucose was increased by nearly 30% with GC treatment (*p* = 0.0505, compared to vehicle) and KRG extract treatment (GC + KRG extract) prevented this increase (*p* = 0.0525 compared to GC + Vehicle). These results indicate that KRG extract treatment may affect GC-induced elevations in blood glucose, a known side effect of chronic GC use. Total femur length was significantly reduced with GC treatment (*p* = 0.0372, compared to vehicle) which was prevented by the KRG extract treatment (GC + KRG extract group).

**TABLE 2:** General body parameters. Values are mean ± SEM. Analysis performed via One-Way ANOVA with Tukey’s posttest. Bolded values are significantly different from Vehicle controls. N = 17-18/group (except for fasting glucose, n = 10). a = *p* < 0.05, b = *p* < 0.01, c = *p* < 0.0001, d = *p* = 0.05.

	Vehicle (n = 18)	Vehicle + KRG extract (n = 18)	GC (n = 18)	GC + KRG extract (n = 17)
Body Weight (g)	35.38 ± 0.78	36.14 ± 0.76	35.04 ± 0.71	33.29 ± 0.60
Liver Weight (g)	1.862 ± 0.08	1.858 ± 0.08	1.898 ± 0.12	1.740 ± 0.05
Total Kidney Weight (g)	0.573 ± 0.018	0.609 ± 0.028	0.581 ± 0.023	0.575 ± 0.014
Spleen Weight (g)	0.09385 ± 0.004	0.09765 ± 0.006	**0.05208 ± 0.003** ^**c**^	**0.0539 ± 0.003** ^**c**^
Visceral Fat (g)	0.764 ± 0.11	0.981 ± 0.11	**1.376 ± 0.11** ^**b**^	1.158 ± 0.16
Fasting Glucose (mg/dL)	286.4 ± 17.0	305.9 ± 15.4	**366.3** ± **31.8** ^**d**^	**286.9 ± 15.1** ^**d**^
Femur Length (mm)	16.07 ± 0.08	15.96 ± 0.10	**15.70 ± 0.11** ^**a**^	15.73 ± 0.09

### KRG extract prevents glucocorticoid-induced trabecular bone loss

**FIGURE 2:**
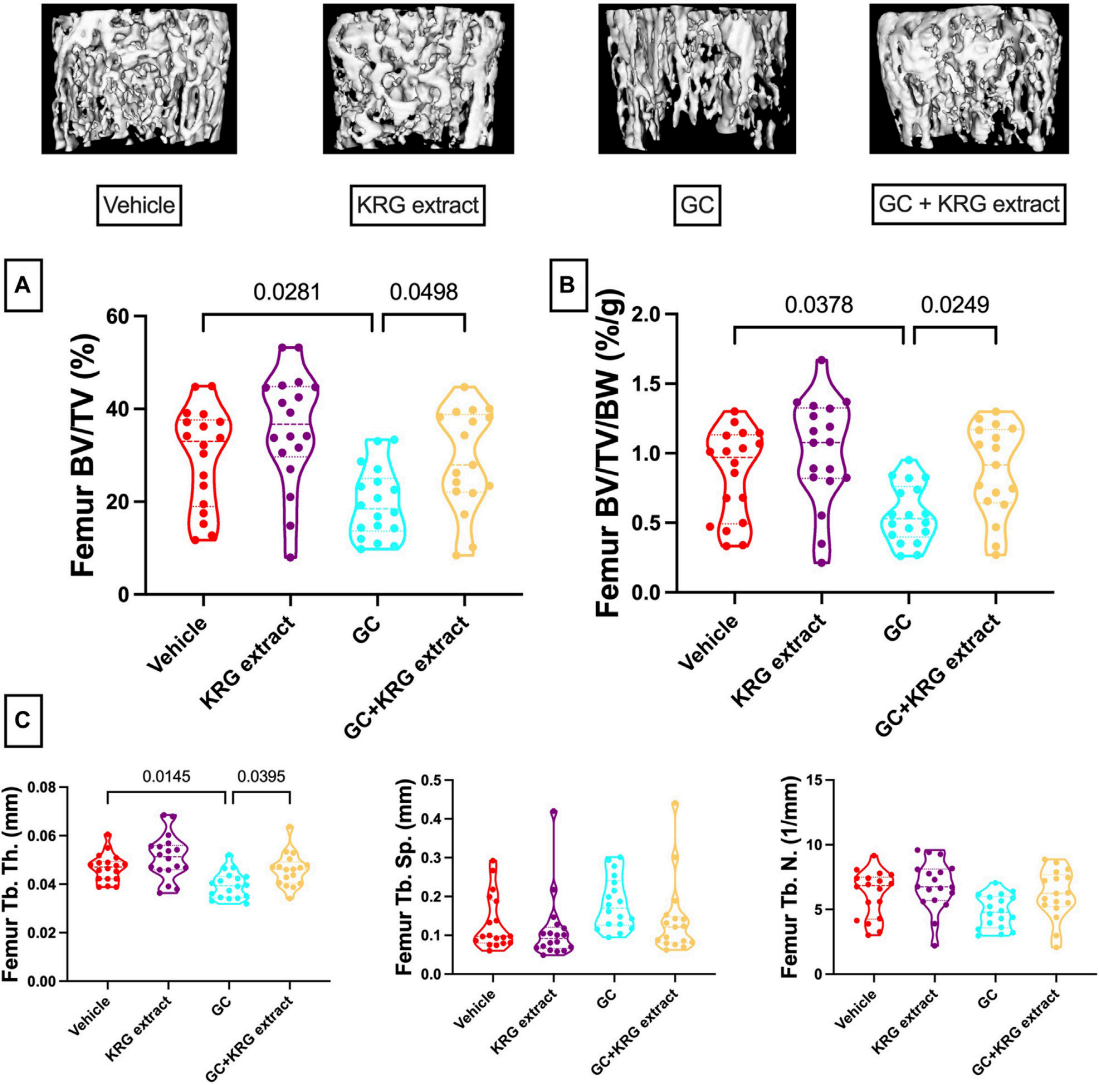
Korean red ginseng extract prevents glucocorticoid induced distal femoral trabecular bone loss in an oral model of glucocorticoid induced osteoporosis in mice. Eight-week-old male CD-1 mice were administered either vehicle or corticosterone in the drinking water for 4 weeks and received vehicle or KRG extract gavage daily for the duration of the study. **(A)** Bone volume/total volume % (BV/TV%) of the distal femur trabecular bone and **(B)** corrected for body weight (BV/TV/BW, %/g). Representative isosurface images of the distal femur trabecular bone are shown above. **(C)** Microarchitectural analyses. Violin plots show the distribution of the data with lines at the median and quartiles. Statistical analyses performed with One-way ANOVA with Tukey’s post-test. n = 17-18/group. Tb. Th. = trabecular thickness, Tb. Sp. = trabecular spacing, Tb. N. = trabecular number.

We next examined GC-induced trabecular bone loss in these groups of mice. As expected, 4 weeks of oral GC administration caused significant trabecular bone loss in the distal femur compared to vehicle controls assessed via BV/TV% (Figure 2A, *p* = 0.0281). Importantly, concurrent administration of KRG extract throughout the 4-week experiment significantly prevented trabecular bone loss induced by GC (GC vs. GC + KRG extract group; *p* = 0.0498). GC + KRG extract group did not differ from Vehicle controls (*p* = 0.9978). Results were similar when BV/TV% was corrected for body weight (Figure 2B). Assessing distal femur trabecular microarchitecture (Figure 2C) revealed that trabecular thickness (Tb. Th.) is significantly reduced with GC treatment (Vehicle vs. GC groups, *p* = 0.0145). Consistent with the effects on BV/TV%, KRG extract treatment significantly prevented GC-mediated decrease in trabecular thickness (GC vs. GC + KRG extract groups, *p* = 0.0395). No significant changes were observed in trabecular spacing (Tb. Sp.) or trabecular number (Tb. N.) with GC and/or KRG extract treatments. Cortical measurements from the mid-diaphyseal region of the femur revealed no major changes in any measures after 4-weeks of GC or KRG extract treatment (Table 3). To understand site-specific effects of GC and KRG extract, we also assessed L4 vertebral trabecular parameters. Although GC decreased vertebral BV/TV % modestly, it was not statistically significant compared to the vehicle group (Vehicle 43.10 ± 1.470 vs. GC 36.11 ± 2.625; Table 4). Interestingly, GC + KRG extract treatment showed a significant increase in BV/TV% (*p* = 0.0111), BV/TV/BW (*p* = 0.0088), and Tb. Th. (*p* = 0.0127, Table 4). compared to the GC group.

**TABLE 3:** Femur mid-diaphyseal cortical parameters. All values are presented as mean ± SEM. Statistical analyses performed via One-Way ANOVA with Tukey’s posttest. Ct. Th. = cortical thickness, Ct. Ar. = cortical area, Ma. Ar. = marrow area, Tt. Ar. = total area, Ec. Pm. = endocortical perimeter, Ps. Pm. = periosteal perimeter.

	Vehicle	Vehicle + KRG extract	GC	GC + KRG extract
(n)	18	18	18	17
Femur Cortical Parameters				
Ct. Th. (mm)	0.301 ± 0.008	0.314 ± 0.006	0.282 ± 0.007	0.292 ± 0.007
Ct. Ar. (mm^2^)	1.323 ± 0.040	1.340 ± 0.029	1.247 ± 0.035	1.264 ± 0.034
Ma. Ar. (mm^2^)	0.977 ± 0.032	0.896 ± 0.034	1.045 ± 0.037	0.952 ± 0.032
Tt. Ar. (mm^2^)	2.300 ± 0.054	2.236 ± 0.050	2.292 ± 0.047	2.216 ± 0.051
Ec. Pm. (mm)	3.674 ± 0.061	3.524 ± 0.068	3.787 ± 0.066	3.617 ± 0.062
Ps. Pm. (mm)	5.561 ± 0.069	5.524 ± 0.072	5.555 ± 0.056	5.475 ± 0.066

**TABLE 4:** L4 vertebral trabecular parameters. All values are presented as mean ± SEM. Statistical analyses performed via One-Way ANOVA with Tukey’s posttest. Bolded values are significantly different from GC group (*p* < 0.05). BV/TV% = bone volume/total volume, BV/TV/BW = bone volume/total volume/body weight, Tb. Th. = trabecular thickness, Tb. Sp. = trabecular spacing, Tb. N. = trabecular number.

	Vehicle	Vehicle + KRG extract	GC	GC + KRG extract
(n)	18	18	18	17
Vertebrae Trabecular Parameters				
BV/TV%	43.10 ± 1.470	46.04 ± 2.019	36.11 ± 2.625	**46.99 ± 3.188**
BV/TV/BW (%/g)	1.231 ± 0.052	1.290 ± 0.071	1.046 ± 0.085	**1.416 ± 0.100**
Tb. Th. (mm)	0.051 ± 0.002	0.052 ± 0.002	0.045 ± 0.003	**0.055 ± 0.003**
Tb. Sp. (mm)	0.067 ± 0.002	0.062 ± 0.004	0.083 ± 0.007	0.066 ± 0.006
Tb. N. (1/mm)	8.554 ± 0.092	8.813 ± 0.208	7.992 ± 0.270	8.404 ± 0.304

### Glucocorticoids impair intestinal barrier integrity

**FIGURE 3:**
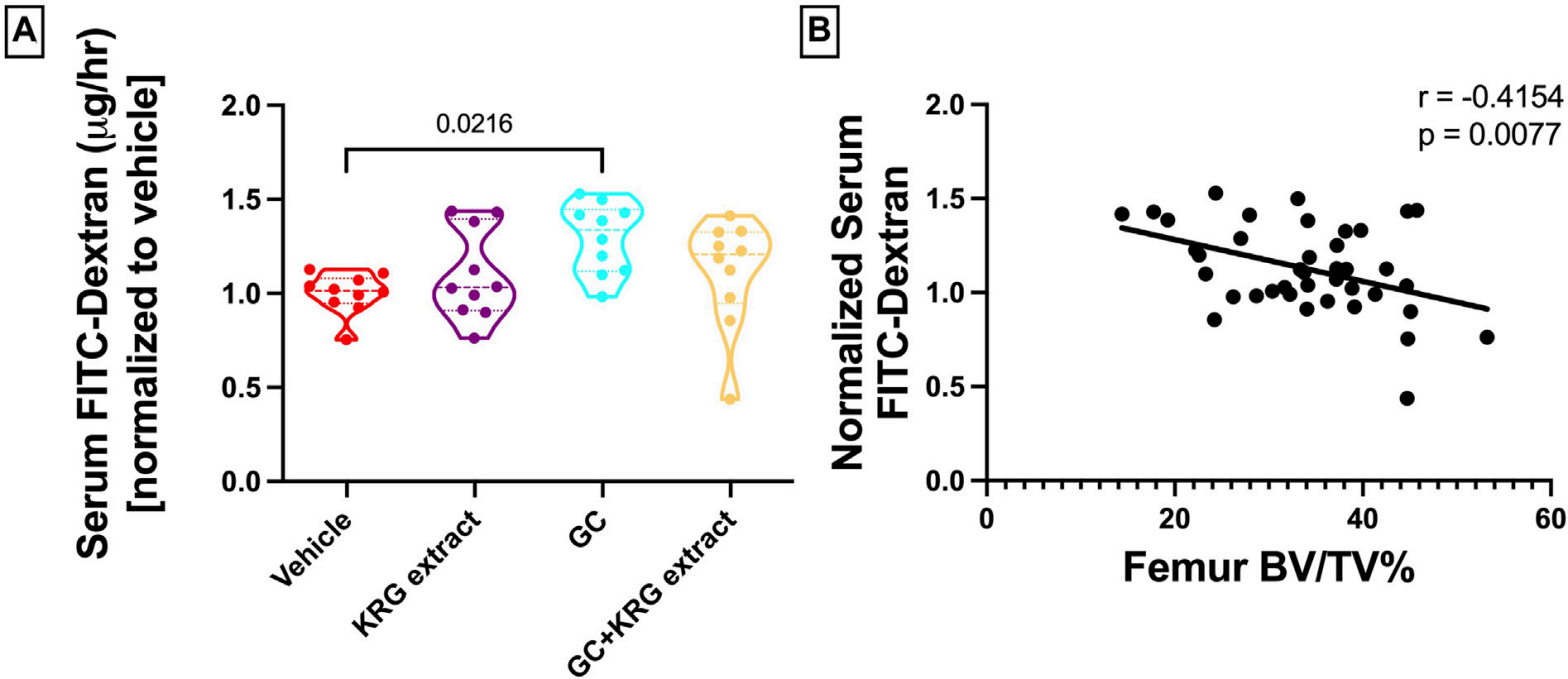
Glucocorticoids increase whole intestinal permeability in CD-1 mice. **(A)** Whole intestinal *in vivo* flux was measured by FITC dextran gavage and subsequent measurement in the serum. **(B)** Pearson’s correlation between distal femur BV/TV% and serum FITC-Dextran. Violin plots show the distribution of the data with lines at the median and quartiles. Statistical analysis performed via One-way ANOVA with Tukey’s post-test. n = 10/group.

We have previously shown that chronic GC treatment impairs intestinal barrier integrity in mice as demonstrated by increased serum endotoxin levels due to leaky gut (Schepper et al., 2020; Chargo et al., 2023). Another method to assess barrier integrity is by measuring serum levels of FITC-Dextran, a method our lab and others have used extensively (Schepper et al., 2019; Behera et al., 2021; Kang et al., 2023). Here, we show that 4-weeks of GC treatment in growing male CD-1 mice increased barrier permeability (*p* = 0.0216) compared to vehicle control as assessed by FITC-Dextran flux into the serum (Figure 3A). KRG extract treatment led to a modest effect (GC + KRG extract group was not statistically different from either GC alone or Vehicle alone groups). KRG extract treatment alone did not affect barrier permeability. Interestingly, there was a significant negative correlation between serum FITC levels and distal femur trabecular bone density (Figure 3B), indicating that impaired barrier function is strongly associated with poor bone health in this model, as we have demonstrated before (Chargo et al., 2023).

### Glucocorticoids and KRG extract alter gut microbiota composition

**FIGURE 4:**
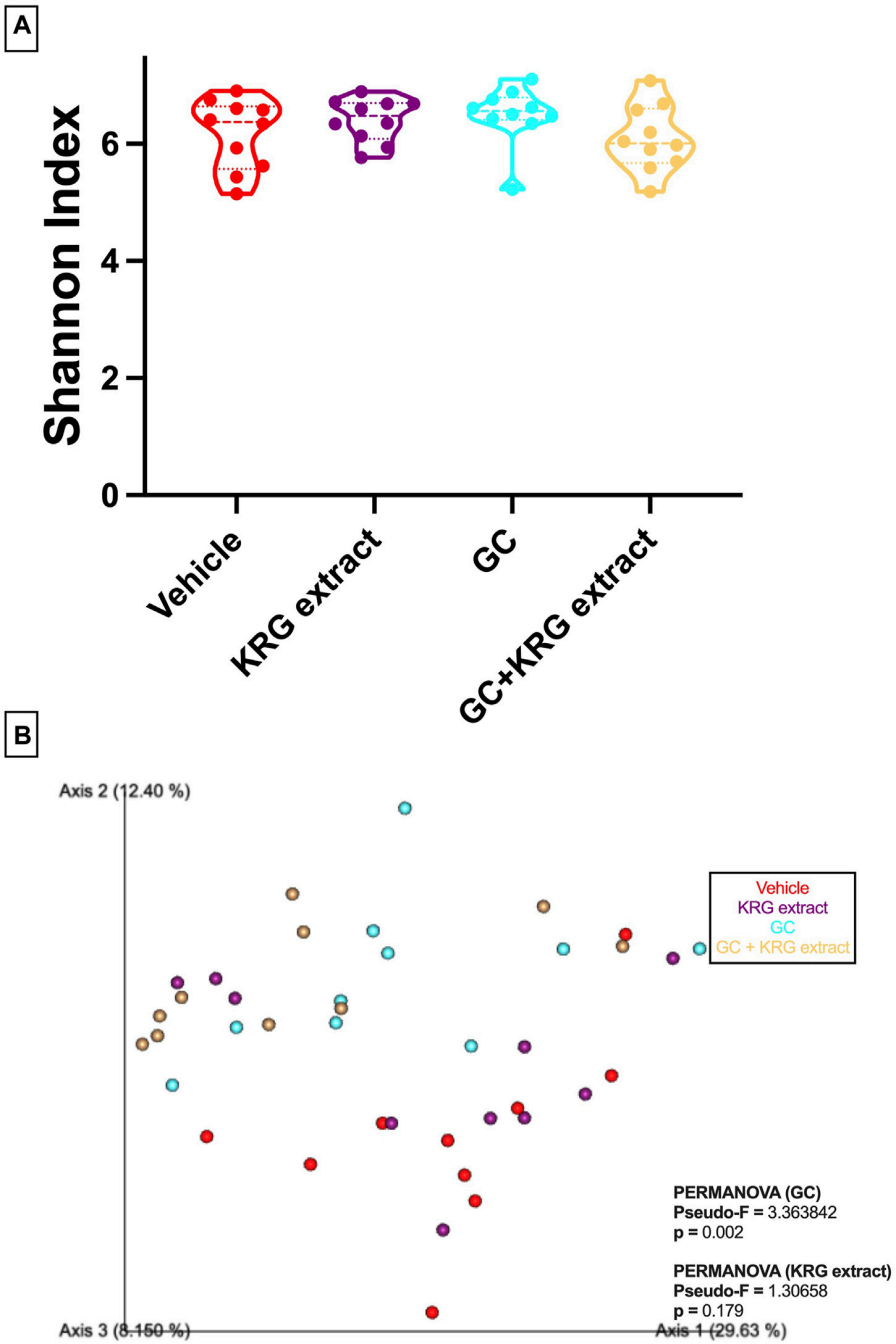
Oral glucocorticoids alter gut microbiota composition in mice. **(A)** Alpha diversity (Shannon index) is unchanged with GC or KRG extract treatment. Analysis via Kruskall-Wallis test with Dunn’s post-test. **(B)** Beta-diversity is altered by 4-week oral GC, but not KRG extract treatment in mice. Principal coordinate analysis plot generated from weighted UniFrac distance matrix. Each dot represents the microbiota composition profile of one mouse. n = 10/group.

**FIGURE 5:**
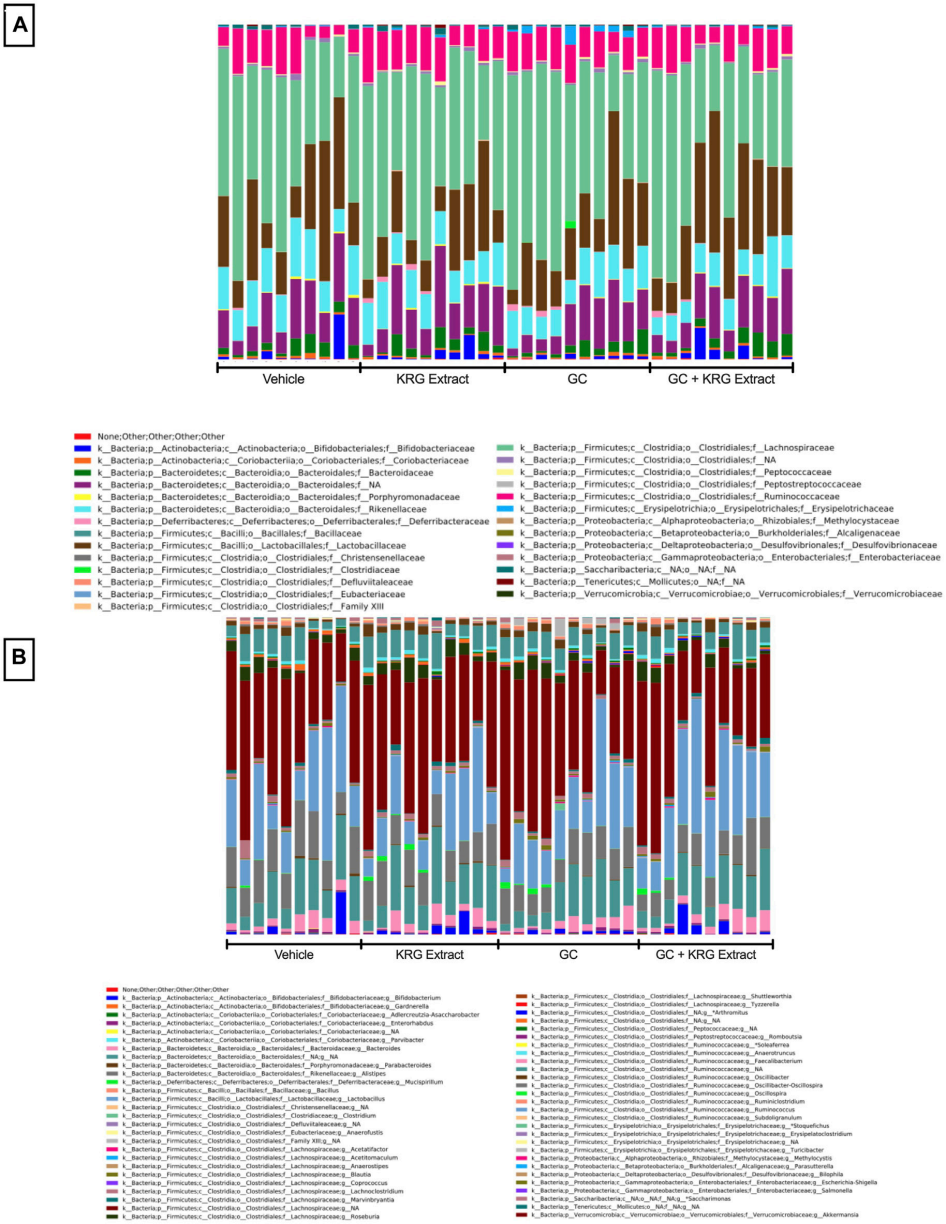
Gut microbiota composition. Stacked bar graphs show relative abundance of individual bacterial taxa at the level of **(A)** family and **(B)** genus. Each bar represents the microbiota composition of one individual mouse following 4-weeks of treatment. Graphs were generated and obtained from Zymo. n = 10/group.

To determine broad effects of GC and KRG extract on microbiota, we examined alpha-diversity (within-sample diversity, or how diverse a single sample is; for example, how many different species are observed in each sample) and beta-diversity (between-sample diversity, or how diverse one sample is compared to another) in fecal microbiota samples. Alpha-diversity as measured by the Shannon Index was not significantly different between the groups with GC or KRG extract treatment (Figure 4A). Beta-diversity was visually represented by principal coordinate analysis plots generated from weighted UniFrac distance matrices. When analyzing all groups, PERMANOVA testing revealed that GC treatment significantly altered microbiota composition after 4-weeks of treatment (Figure 4B, *p* = 0.002) while KRG extract did not cause any significant changes (*p* = 0.179). Upon further analysis between groups, KRG extract modestly altered the microbiota composition within the GC treated groups (GC vs. GC + KRG extract; *p* = 0.103). KRG treatment alone did not affect beta-diversity (*p* = 0.417). To visualize relative abundance differences in microbiota composition between groups, stacked bar graphs were generated at the taxonomic level of family (Figure 5A) and genus (Figure 5B). Each individual bar represents the unique microbiota composition from one mouse at the end of the study. At the family level, the microbiota composition appears to be dominated by family *Lachnospiraceae* and family *Lactobacillaceae* (Figure 5A). At the genus level, the microbiota composition appears to be dominated by an undefined genus within the family *Lachnospiraceae* and genus *Lactobacillus* (Figure 5B).

**FIGURE 6:**
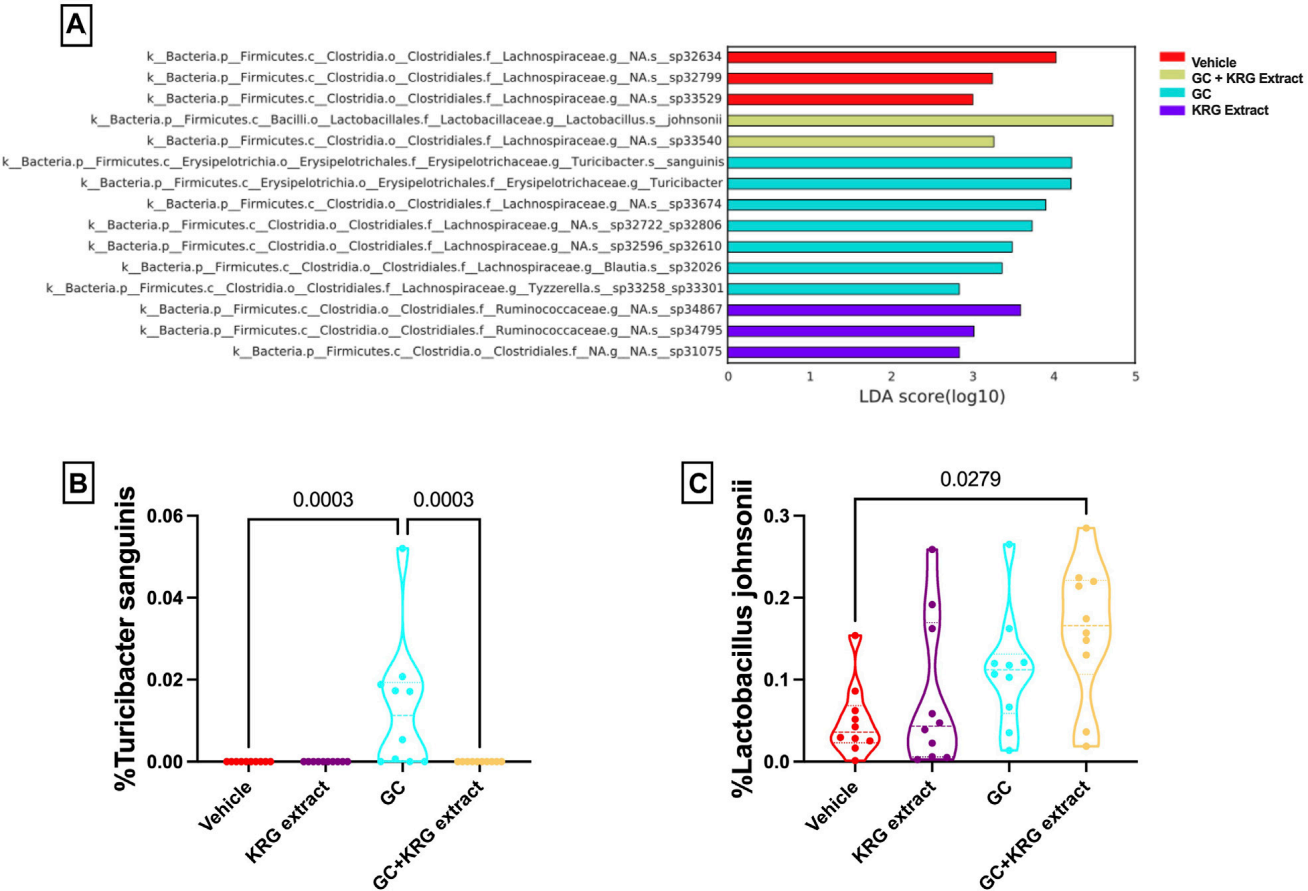
Glucocorticoids and Korean Red Ginseng extract cause species level alterations in the composition of the gut microbiota. **(A)** LEfSe analysis to determine species level changes in gut microbiota composition. Differences in percent relative abundance of **(B)** *Turicibacter sanguinis* and **(C)** *Lactobacillus* johnsonii after 4 weeks of treatment. LEfSe analysis performed as described in methods section. Violin plots show the distribution of the data with lines at the median and quartiles. Group differences in specific microbial species determined via Kruskall-Wallis test with Dunn’s multiple comparisons posttest. n = 10/group.

While broad level analyses (alpha- and beta-diversity) revealed some differences in the composition of the microbiota, examining the microbiota composition at a deeper level (i.e., species) revealed several significant differences between groups, providing further information about specific changes resulting from GC or KRG extract treatment. Linear discriminant analysis effect size (LEfSe) analyses were performed to determine which specific bacterial organisms had the potential to explain differences between groups. LEfSe identifies specific organisms that are significantly different between groups (*p* < 0.05) and calculates effect sizes (linear discriminant analysis, LDA scores) to provide insight into which bacterial organisms may be driving group differences in detail at the species level. Figure 6A shows results from LEfSe analysis with larger LDA scores indicating larger effect sizes. The chart is color coded to indicate which group the bacterial organism had the highest abundance in. Several of the bacterial organisms that fit the criteria of significant group differences (*p* < 0.05) and LDA score >2 are unknown species. However, two named species were present in the final LEfSe analysis. Of particular interest, *Turicibacter sanguinis* was only detected in the GC group (Figure 6B) and strikingly different from GC + KRG extract treated group, indicating that it may be an important organism driving changes in microbiota composition in the GC group. Consistent with GC’s effect on *T. sanguinis* and KRG extract’s ability to reverse it, spearman correlation of *T. sanguinis* with distal femur trabecular BV/TV% reveals a significant negative correlation (r = −0.515, *p* = 0.0007), indicating that increased levels of this bacteria associate with poor bone health. In addition to the relationship to bone health, there is a significant positive correlation between *T. sanguinis* and serum FITC levels (r = 0.463, *p* = 0.003) indicating that increased levels of this bacteria associate with impaired gut barrier function. Additionally, *Lactobacillus johnsonii* significantly differed between groups with an increase in the GC + KRG extract group compared to vehicle controls (Figure 6C, *p* = 0.0279).

**FIGURE 7:**
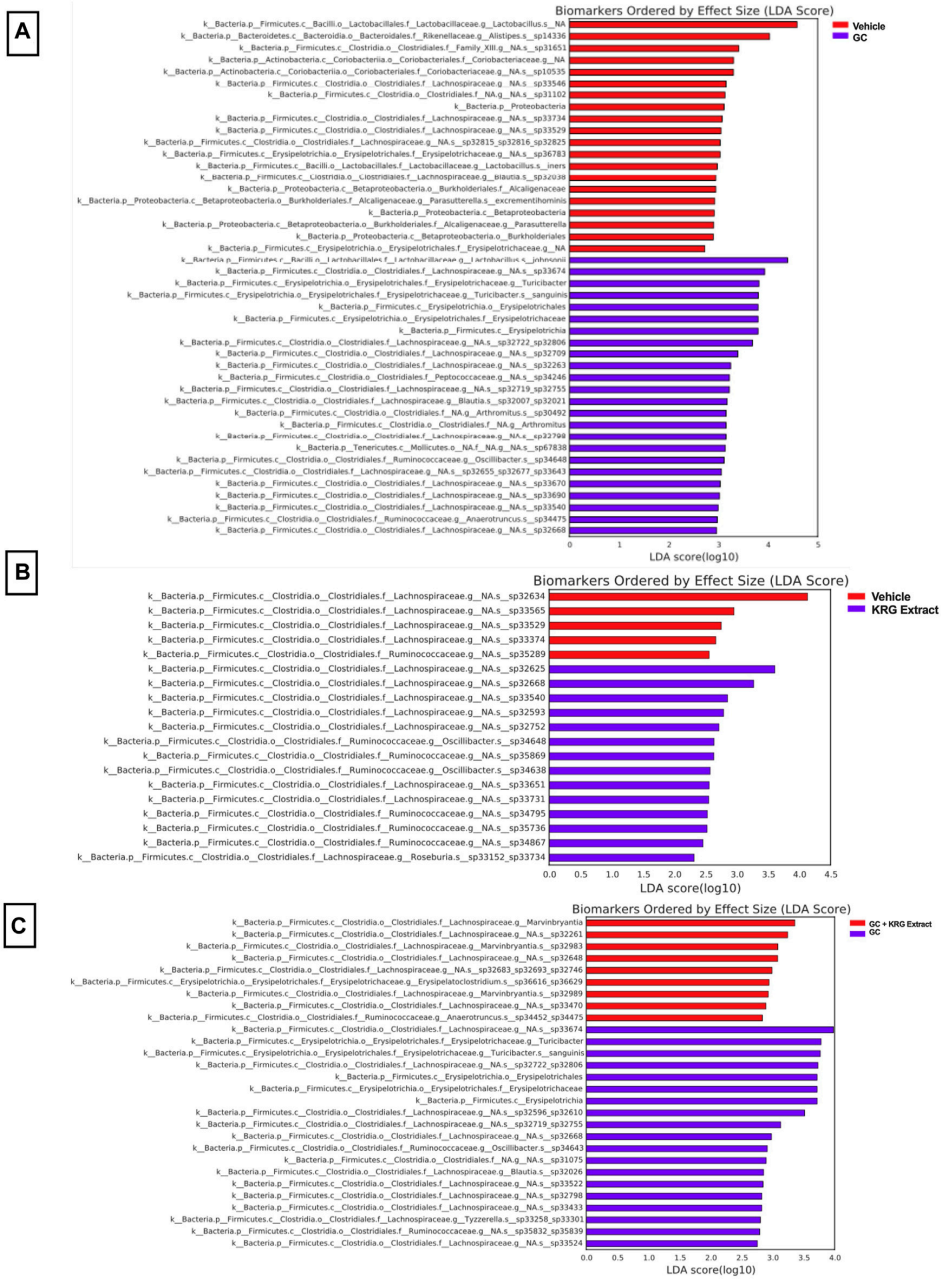
Individual bacterial taxa may serve as biomarkers for driving responses to treatment. LEfSe analysis of the gut microbiota after 4-weeks of treatment. Each table represents comparisons between two groups: **(A)** vehicle vs. GC, **(B)** vehicle vs. KRG extract, and **(C)** GC vs. GC + KRG extract. Colored bar corresponds to key associated with each table. n = 10/group.

To gain further insight into which bacteria were driving differences between groups, LEfSe analysis was also conducted on specific comparisons between groups: Vehicle vs. GC (Figure 7A), Vehicle vs. KRG Extract (Figure 7B), and GC vs. GC + KRG Extract (Figure 7C). These specific paired comparisons generated slightly different effect size results compared to analysis conducted on all groups (Figure 6A). In the Vehicle vs. GC comparison, the taxa with the largest effect size in each group were an unidentified species within genus *Lactobacillus* (LDA = 4.58) for the vehicle group and *Lactobacillus johnsonii* (LDA = 4.39) in the GC group (Figure 7A). In the Vehicle vs. KRG Extract comparison, the taxa with the largest effect size in each group were an unidentified species and genus within family *Lachnospiraceae* (LDA = 4.13) for the vehicle group and a different unidentified species and genus within family *Lachnospiraceae* (LDA = 3.61) in the KRG Extract group (Figure 7B). In the GC vs. GC + KRG Extract comparison, the taxa with the largest effect size in each group were an unidentified species and genus within family *Lachnospiraceae* (LDA = 3.98) in the GC group and genus *Marvinbryantia* (LDA = 3.36) in the GC + KRG Extract group (Figure 7C). These results provide further insight into which bacteria may be driving differences between groups resulting from different treatments and warrant further investigation as potential biomarkers.

### Glucocorticoids and KRG extract alter immune cell populations in the bone marrow and mesenteric lymph node

**FIGURE 8:**
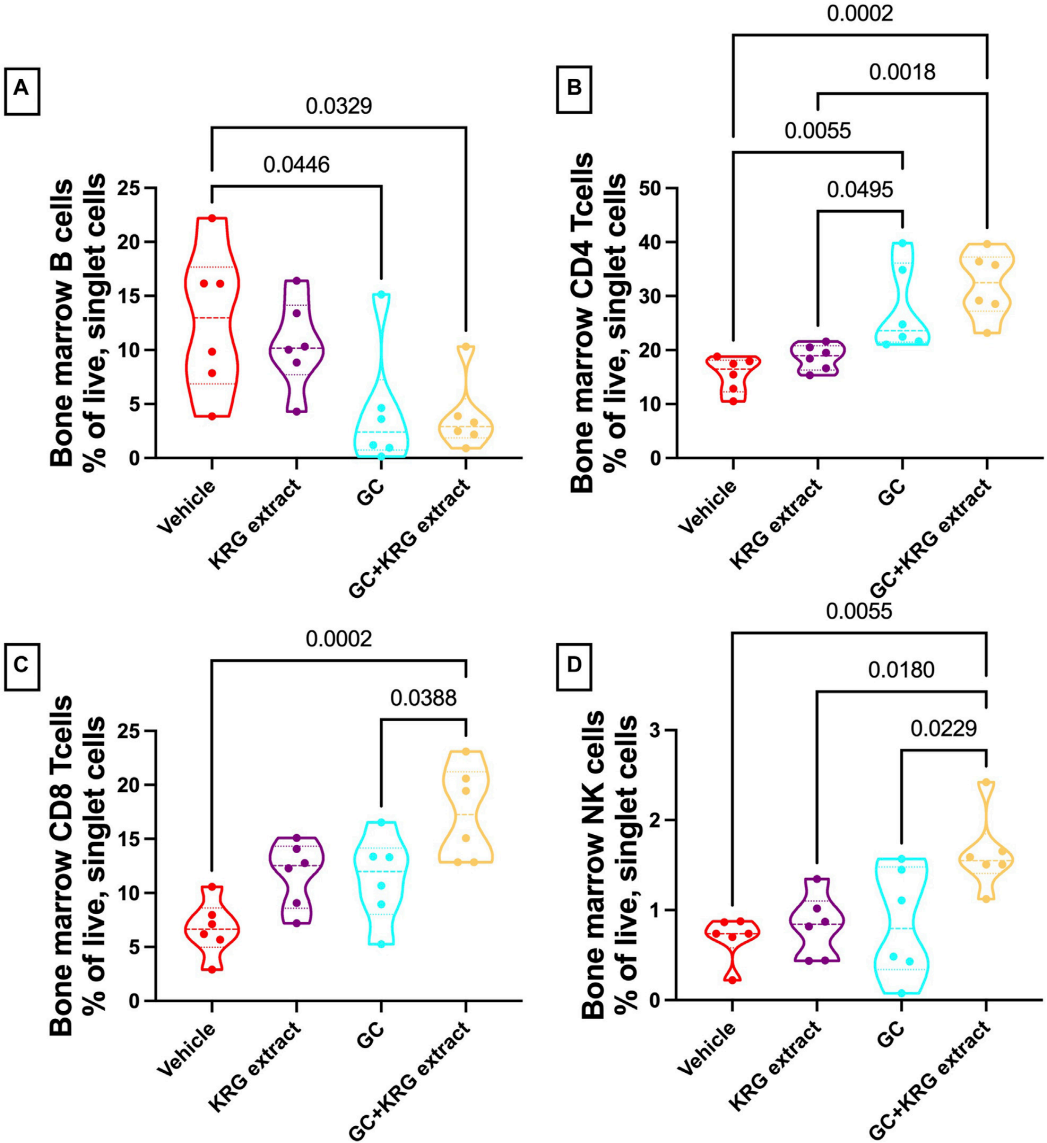
Glucocorticoids and KRG extract alter multiple immune cell populations in the bone marrow. Bone marrow immune cell populations were analyzed after 4 weeks of treatment via flow cytometry. Differences in **(A)** B-cells, **(B)** CD4^+^ T-cells, **(C)** CD8^+^ T-cells, and **(D)** NK cells were analyzed via One-Way ANOVA with Tukey’s posttest. Violin plots show the range of values with lines at the median and quartiles. n = 5-6/group.

To assess immune cell population changes with GC and KRG extract treatments, bone marrow (femur) and mesenteric lymph nodes were collected during harvest and assessed for B- and T-lymphocytes and NK cells using flow cytometry. Analysis of the bone marrow cells revealed that B-cells were reduced in the GC group and that KRG extract treatment (GC + KRG extract group) did not affect GC-mediated decrease of B-cells (*p* < 0.05, compared to vehicle, Figure 8A). KRG extract treatment alone did not cause any significant changes. Interestingly, bone marrow CD4^+^ T-cells were increased with GC treatment and KRG extract treatment (GC + KRG extract group) did not affect this increase (*p* < 0.01, compared to vehicle, Figure 8B). KRG extract treatment alone did not have any effect. Although CD8^+^ T-cells in the bone marrow were not affected by either KRG extract alone or GC alone, KRG extract treatment in the presence of GC (GC + KRG extract) significantly increased CD8^+^ T cells (*p* = 0.0002, compared to vehicle; *p* = 0.0388, compared to GC; Figure 8C). Similar results were seen in bone marrow NK cells, with GC + KRG extract treatment being increased compared to both Vehicle (*p* = 0.0055) and GC groups (*p* = 0.0229, Figure 8D).

**FIGURE 9:**
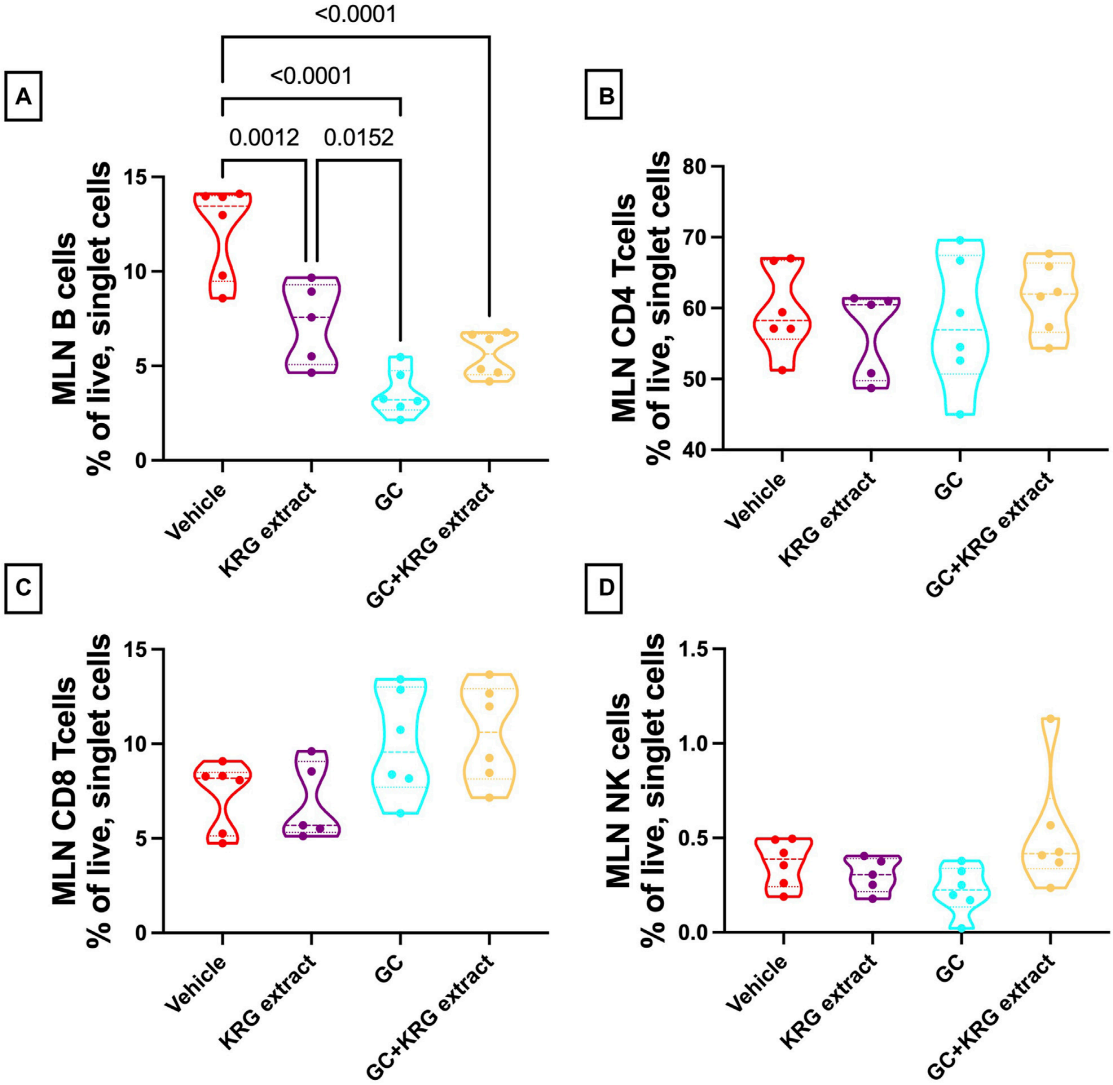
Glucocorticoids and KRG extract alter B-cell populations in the mesenteric lymph node. Mesenteric lymph node immune cell populations were analyzed after 4 weeks of treatment via flow cytometry. Differences in **(A)** B-cells, **(B)** CD4^+^ T-cells, **(C)** CD8^+^ T-cells, and **(D)** NK cells were analyzed via One-Way ANOVA with Tukey’s posttest. Violin plots show the range of values with lines at the median and quartiles. n = 5-6/group.

Analysis of the mesenteric lymph node demonstrated a significant reduction in B-cells with GC treatment and KRG extract did not affect this decrease, similar to bone marrow (*p* < 0.0001, compared to vehicle; Figure 9A). Interestingly, KRG treatment alone caused a significant reduction in B-cells compared to Vehicle control (*p* = 0.0012, Figure 9A). Further analyses in the mesenteric lymph node immune cell populations revealed no differences between any groups in CD4^+^ T-cells (Figure 9B), CD8^+^ T-cells (Figure 9C), or NK cells (Figure 9D).

## Discussion

The goal of the current study was to investigate if Korean Red Ginseng (KRG) extract could prevent bone loss in a clinically relevant, oral model of glucocorticoid-induced osteoporosis (GIO) in male outbred CD-1 mice. Additionally, we wanted to better understand potential mechanisms by which KRG extract affects the gut-bone axis in this model. The gut-bone axis has garnered much attention over the past decade and it is well accepted that the intestinal microbiota plays a pivotal role in controlling bone homeostasis. Several seminal studies from our lab and others have demonstrated that alterations to the gut microbiome or direct targeting of the microbiome can modulate bone density in germ-free mice (Sjögren et al., 2012; Quach et al., 2018), prevent primary osteoporosis in rodents and humans (Britton et al., 2014; Ohlsson et al., 2014; Li et al., 2016; Nilsson et al., 2018), prevent diabetes induced bone loss (Zhang et al., 2015), prevent post-antibiotic dysbiosis induced bone loss (Schepper et al., 2019), and importantly in the context of this study, GIO (Schepper et al., 2020). Previous studies have demonstrated a protective role of KRG extract in several etiologies of bone loss (Siddiqi et al., 2013; Kang et al., 2020; 2023). Kim et al. previously reported that KRG extract dosed at 100 and 500 mg/kg/day effectively prevented GIO in a sub-cutaneous GC pellet model (Kim et al., 2015). However, they did not investigate the role of KRG extract in modulating the gut-bone axis. Using the subcutaneous pellet model, we have shown that chronic GC leads to significant trabecular bone loss, gut microbiota dysbiosis, and impaired gut barrier function (Schepper et al., 2020). We also showed that gut microbiota and intestinal barrier are important therapeutic targets to prevent bone loss in the GIO model. Even though the subcutaneous model mimics the human bone response well, the route of drug delivery is different from the oral route commonly used in humans. To address this difference, our lab and others have recently utilized a clinically relevant model of GIO in growing mice that has an oral delivery route via the drinking water (Gasparini et al., 2016; Chargo et al., 2023). The benefits of using this model are that it mimics the drug delivery system in human therapeutics and also allows for direct physical interaction between the glucocorticoid (GC), gut microbiota, and gut barrier, as well as gut targeted therapies (KRG extract in this case). Additionally, the model uses CD-1 mice which are outbred and have more genetic diversity akin to humans, thus preventing possible treatment effects due to clonal genetics. Using this model, we recently showed that probiotic *Lactobacillus reuteri* 6475 can prevent GC-induced gut barrier dysfunction as well as bone loss (Chargo et al., 2023). In the current study, we treated mice with 500 mg/kg/day KRG extract along with oral GC and observed that trabecular bone loss was significantly prevented along with changes to gut microbiota composition, providing evidence for the first time that KRG extract influences the gut-bone axis in the oral GC-induced GIO model.

Glucocorticoids are used clinically for their anti-inflammatory effects. However, their effect on the immune system, especially in healthy CD-1 mice, has not been investigated before. Our findings indicate that oral GC has distinct as well as overlapping effects on immune population in the mesenteric lymph node (MLN) and bone marrow. In both sites, GC suppressed B cell population and importantly, KRG extract did not affect this decrease, suggesting that KRG’s effect on bone is likely independent of GC’s effect on B cells. Compared to B cells, GC did not affect T cells or NK cells in the mesenteric lymph node. However, GC had variable effects on these cell types in the bone marrow. CD4^+^ T cells were increased with GC and KRG extract did not affect this increase. Interestingly, although CD8^+^ T cells and NK cells were not affected by GC, concurrent KRG extract treatment enhanced these cells even though KRG alone did not affect these cells. The significance of these findings is not clear especially in the context of bone health. It is important to note, however, that CD8^+^ T cells in the bone marrow are an important source of Wnt10b, a critical regulator of bone density through its effect on osteoblastogenesis (Terauchi et al., 2009). Therefore, it is possible that KRG extract influences CD8^+^ T cells to positively influence bone health. On the other hand, it is not clear if the increase in these cells affect the anti-inflammatory effect of GC. Further studies will be needed in inflammatory disease models to understand if KRG extract influences bone health without affecting GC’s anti-inflammatory effects.

Several studies have previously examined the effect of KRG extract on the gut microbiota and its association with several health conditions (Chen et al., 2022). In a recent study (Kang et al., 2023), we demonstrated that KRG extract can significantly modulate gut microbiota and the gut-bone axis in an antibiotic-induced dysbiosis model. We found that KRG extract modulated several aspects of the gut microbiota in not only naïve healthy mice but also in antibiotic-induced dysbiotic mice. For example, we observed changes in relative abundance of *Lactobacillus*, rc4-4, and an unnamed genus in the family S24-7 that correlated with distal femur trabecular bone volume. Interestingly, our lab and others have previously reported beneficial effects on bone (and gut) health through supplementation of probiotic *Lactobacillus reuteri* 6475 in several etiologies of bone loss (McCabe et al., 2013; Britton et al., 2014; Zhang et al., 2015; Collins et al., 2016; 2019; Quach et al., 2019; Schepper et al., 2019; 2020; Rios-Arce et al., 2020). In the current study, we observed that *Lactobacillus johnsonii*, was significantly increased in the GC + KRG extract group compared to the vehicle control group. To our knowledge, there are no reports linking *Lactobacillus johnsonii* to bone density in humans or rodents. However, a recent study demonstrates that treating mice with a multi-herbal supplement containing panax ginseng (BaWeiBaiDuSan) increases the abundance of *Lactobacillus johsonii* and alleviates polymicrobial sepsis-induced liver injury in mice (Fan et al., 2023). In addition, treatment with *Lactobacillus johnsonii* also prevents polymicrobial sepsis-induced liver injury and this was associated with an increase in serum IL-10 suggesting that *L. johnsonii* may have an overall anti-inflammatory effect. Along with the evident anti-inflammatory effect, *Lactobacillus johnsonii* has also been implicated in the prevention of intestinal barrier dysfunction in several models (Chen et al., 2021; Wan et al., 2022; Bai et al., 2023; Lyu et al., 2023). This has been evidenced by improvements in tight junction proteins such as ZO-1, occludins, and claudin-1, along with reductions in circulating D-lactate, a systemic marker used to investigate intestinal barrier function. Future studies will be necessary to further test the direct role of *L. Johnsonii* on both gut and bone health in this oral model of GIO.

Another species that was significantly modulated by GC treatment was *Turicibacter sanguinis*. Interestingly, this species was present only in the GC treated group and was absent in the GC + KRG extract treated groups. *T. Sanguinis* phylogenetically belongs to phylum Firmicutes, class Erysipelotrichia, order Erysipelotrichales, and family *Erysipelotrichaceae*. To the best of our knowledge, *T. sanguinis* has not been directly tested in the context of bone health or gut barrier function. In a study using a transverse aortic constriction model, *T. sanguinis* was significantly elevated following disease induction and was strongly associated with impaired intestinal barrier function in mice, indicated by reductions in colon occludin mRNA as well as increased serum FITC in the disease group (Boccella et al., 2021). The negative association between *T. Sanguinis* and bone BV/TV% and positive association with serum FITC levels suggests that *T. sanguinis* could be a biomarker for poor bone health and gut barrier function in this model. Interestingly, *T. sanguinis* was shown to be responsible in part for regulating peripheral serotonin production in the intestine (Fung et al., 2019). Several studies have established that peripheral serotonin production has a negative effect on osteoblast function by inhibiting bone formation (Ducy and Karsenty, 2010; Yadav et al., 2010). In addition, peripheral serotonin production has been linked with impaired gut barrier function (Yamada et al., 2003; Rosa et al., 2023). Together, these findings could suggest that increased *T. sanguinis* (only present in the GC group) leads to increased peripheral serotonin production in the gut, which then leads to impaired gut barrier function and inhibition of bone formation; while KRG extract treatment ablates the increase in *T. sanguinis*, preventing the deleterious effects associated with increased *T. sanguinis*. Future studies will investigate the role of *T. sanguinis* and peripheral serotonin production and the effectiveness of *T. sanguinis* as a biomarker in this and other models or if this would be translatable to humans.

The results of the LEfSe analysis demonstrate the complex nature of the gut microbiota and the need for further research to describe its composition and complex relationships within. It also demonstrates the necessity for deep level, specific analyses when conducting these types of studies. For example, when comparing the Vehicle vs. KRG Extract groups, there are two unique, unidentified species and genus within the family of *Lachnospiraceae* with the largest effect size in each group, thus indicating that each plays the largest role in driving the changes seen within that specific group. This demonstrates how within families of bacteria, specific taxa may have differential effects and can potentially be driving different changes in bacterial community composition. As such, the need for specific analyses at the deepest taxonomic level is evident and should be pursued.

Taken together, our study demonstrates for the first time that KRG extract significantly affects gut-bone axis in the context of oral GC treatment in CD-1 mice. Our studies suggest that concurrent oral ingestion of KRG extract during oral GC treatment in humans may be beneficial for bone health. Future clinical trials are needed to confirm these findings in humans.

## Data Availability

The 16S sequencing data presented in this study are publicly available at EBI ENA, accession numbers PRJEB72124 ERP156904. Study information is available at https://qiita.ucsd.edu/public/?study_id=15017, Study ID 15017; with subsequent analysis at https://qiita.ucsd.edu/analysis/description/57462/, Analysis ID 57462.
